# Repair of a “long and narrow” skin defect of the upper extremity with a modified design of a compound SCIP flap: a series of 12 cases

**DOI:** 10.1186/s40001-024-01863-y

**Published:** 2024-05-09

**Authors:** Haiwen Wang, Zetian Shi, Deqing Zeng, Haibo Wang, Pengcheng Lv, Pei Li

**Affiliations:** Department of Hand Surgery, Dongguan Chashan Hospital, Guangdong Medical University, Dongguan, 523000 China

**Keywords:** Conjoined perforator flap, Free inguinal flap, Superficial circumflex iliac artery(SCIA), Posterior intercostal arteries perforator (PICAP), Lumbar artery perforator flaps (LAPs), Upper extremity reconstruction

## Abstract

**Background:**

Large skin lesions of the upper extremity tend to be ‘‘long and narrow’’ in shape, and the currently used repair and reconstruction protocols still have some drawbacks, including difficulty in closure of the donor area, poor cosmetic appearance of the donor and recipient areas, and low flap survival rates. The ilioinguinal flap has been more widely used for repair and reconstruction of various complex conditions. In order to improve the versatility of the flap design and to achieve better aesthetic results, we report a study on the improved design of Compound SCIP flap for repairing "long and narrow" large skin defects of the upper extremity by using a modified design of the ilioinguinal flap for the procurement of perforating blood vessels and flap excision.

**Methods:**

From April 2005 to August 2015, a total of 12 patients underwent this modified design procedure, in which the anterior branch of the fourth lumbar artery or the posterior intercostal artery was selected to provide blood supply for the perforator flap together with the superficial branch of the superficial iliac artery to meet the blood supply needs of the flap for the one-time repair of a large "long and narrow" skin defect in the upper limb. Patient demographics, flap characteristics, and associated complications were retrospectively analyzed.

**Results:**

3 females and 9 males were included in this study, the mean age of the patients was 31.7 years (range, 22–44 years), the mean follow-up period was 15.3 ± 5.6 months (range, 7–24 months), and all patients had complete closure of the defect site and donor area, and all flaps survived.

**Conclusions:**

The Compound SCIP flap presents some advantages in repairing 'long and narrow' skin defects in the upper limb. While ensuring the survival rate of the elongated ilioinguinal flap, it amplifies the benefits of the ilioinguinal flap and enhances skin utilization. This can serve as a beneficial choice for repairing 'long and narrow' skin defects in the upper limb.

**Supplementary Information:**

The online version contains supplementary material available at 10.1186/s40001-024-01863-y.

## Background

Given the extended longitudinal axis inherent to the upper limb, extensive soft tissue defects frequently manifest in narrow and elongated configurations. Traditional approaches to repair such defects utilizing large skin flaps often encounter limitations, particularly in the direct suturing of the donor area post-repair. Consequently, secondary grafting or supplementary flap coverage becomes imperative. This escalation of intervention not only amplifies the healing demands on the patient but also engenders increased surgical procedures, prolonged hospitalization, and financial burden. Furthermore, maintaining a satisfactory aesthetic outcome in the donor area poses a formidable challenge within this paradigm [1–3]. With the evolution of microsurgical techniques, the pursuit of achieving meticulous closure of the donor area and optimal aesthetic outcomes in flap reconstruction has gained significant traction. The ideal reconstructive approach aims to achieve superior results while minimizing surgical trauma. In this context, the utilization of the ilioinguinal flap sourced from the groin region emerges as an advantageous option. This flap, characterized by its hairless nature, appropriate thickness, favorable donor site scarring, high vascular reliability, and procedural simplicity, presents notable benefits. Typically vascularized by the superficial circumflex iliac artery (SCIA), a branch of the femoral artery, the ilioinguinal flap offers a reliable vascular supply. However, in scenarios necessitating extensive soft tissue reconstruction, particularly for ‘‘long and narrow’’ wound defects, the limitations of a freestyle flap relying on a singular arterial supply may become apparent. This constraint may impede the ability to harvest a sufficient area while ensuring flap viability, thereby potentially compromising the reconstructive outcome [4–8]. To optimize the benefits associated with the ilioinguinal flap and enhance skin utilization, we have innovatively designed a compound perforator flap based on the ilioinguinal region. This study delineates our experience and treatment outcomes in utilizing the compound SCIP flap for the transplantation and reconstruction of extensive soft tissue defects in the upper limb. This flap variant is comprised of branches originating from the SCIA, supplemented by either the anterior branch of the fourth lumbar artery (L4 artery) or posterior intercostal artery (PICA).

## Methods

We conducted a retrospective chart review of 12 patients who underwent ilioinguinal composite perforator flap treatment between April 2005 and August 2015 (Table 1 and 2). The recorded data encompassed demographic characteristics, etiology, defect sizes, flap specifics, surgical details, postoperative complications, specific choices for vascular anastomosis, and types of flap perforators.

**Table 1:** Case summary and characteristics

No	Age	Sex	Weight (kg)	Etiology	Defect size (cm)	Flap size (cm)	Flap harvest time (min)	Secondary thinning surgery	Flap survival	Donor site complication / post-operative complications	Follow-up (month)
1	25	male	67	hot-press	23 × 8	25 × 9	82	no	yes	none	12
2	28	Male	71	Crush	26 × 10	28 × 11	92	No	Yes	None	24
3	22	Female	52	Contusion/abrasion	29 × 10	30 × 11	116	No	Yes	None	21
4	41	Male	65	Traffic accident	27 × 12	29 × 13	112	No	Yes	None	10
5	31	Male	71	Hot-press	31 × 13	33 × 14	108	No	Yes	None	15
6	29	Male	63	Crush	33 × 14	33 × 15	104	Yes	Yes	None	21
7	36	Female	55	Avulsion	25 × 8	27 × 9	86	No	Yes	None	8
8	27	Female	59	Crush	28 × 8	29 × 9	91	No	Yes	None	22
9	34	Male	69	Traffic accident	30 × 9	31 × 10	102	No	Yes	None	11
10	44	Male	76	Hot-press	28 × 11	30 × 12	112	No	Yes	Hematoma	7
11	39	Male	75	Crush	32 × 10	34 × 11	107	Yes	Yes	None	18
12	24	Male	66	Avulsion	34 × 12	35 × 15	115	No	Yes	None	14
Mean	31.7	–	65.8	–	28.8 × 10.4	30.3 × 11.6	102.3	–	–	–	15.3
SD	6.8	–	7.2	–	3.2 × 1.9	2.7 × 2.1	11.2	–	–	–	5.6

SD, standard deviation

**Table 2:** Perforator type and anastomosis selection

No	Arterial vessel anastomosis	Venous vessel anastomosis	Perforator type
1	SCIAs—PIA SCIA—PIA L4 segmental artery—Dorsal carpal branch of the radial artery	AV-SCIA—PIAV SCIV—CV	SCIP-LAP
2	SCIAs—BA PICA—SBRA	AV-SCIA—AV-BA SCIV—Upper arm subcutaneous vein	SCIP-PICAP
3	SCIA—BA PICA—RA	AV-SCIA—AV-BA SCIV—Upper arm subcutaneous vein	SCIP-PICAP
4	SCIA—PIA L4 artery—SBRA	AV-SCIA—PIAV SCIV—CV	SCIP-LAP
5	SCIA—PIA L4 artery—Dorsal carpal branch of the radial artery	AV-SCIA—PIAV SCIV- CV	SCIP-LAP
6	SCIA—PIA L4 artery—Dorsal carpal branch of the radial artery	AV-SCIA—PIAV SCIV—CV	SCIP-LAP
7	SCIA—BA PICA—RA	AV-SCIA—AV-BA SCIV—Upper arm subcutaneous vein	SCIP-PICAP
8	SCIA—BA PICA—RA	AV-SCIA—AV-BA SCIV—Upper arm subcutaneous vein	SCIP-PICAP
9	SCIA—BA PICA—RA	AV-SCIA—AV-BA SCIV—Upper arm subcutaneous vein	SCIP-PICAP
10	SCIA—PIA L4 artery—Dorsal carpal branch of the radial artery	AV-SCIA—PIAV SCIV—CV	SCIP-LAP
11	SCIA—PIA L4 artery—Dorsal carpal branch of the radial artery	AV-SCIA—PIAV SCIV—CV	SCIP-LAP
12	SCIA—PIA L4 artery—Dorsal carpal branch of the radial artery	AV-SCIA –PIAV SCIV—CV	SCIP-LAP

SCIA superficial circumflex iliac artery, SCIAs superficial branch of superficial circumflex iliac artery, *AV-SCIA* accompanying vein of the superficial branch of the circumflex iliac artery, *SCIV* superficial circumflex iliac vein, *PIA* posterior interosseous artery, *PIAV* vein accompanying the posterior interosseous artery, *RA* radial artery, *BA* brachial artery, *AV-BA* accompanying vein of the brachial artery, *CV* cephalic vein, *SBRA* superficial branch of the radial artery, *RA* radial artery, *PICA* posterior intercostal artery, *SCIV* superficial circumflex iliac vein

## Surgical techniques

Patients were placed in a supine position and administered general anesthesia prior to the initiation of surgical intervention. In instances characterized by substantial soft tissue deficits following trauma, the initial approach encompassed comprehensive wound management, entailing meticulous debridement of necrotic and nonviable tissues. Employing surgical microscopy to optimize visualization, a meticulous dissection was performed to identify and safeguard perforator arteries deemed suitable for anastomosis. These identified arteries were carefully delineated to facilitate their preservation and subsequent incorporation into the reconstruction procedure.

The preoperative planning phase involved the utilization of a handheld Doppler ultrasound device to delineate the surface projection areas adjacent to the superficial branches of key arteries, namely the SCIA, the anterior branch of the L4 artery, and the PICA. The exit points of these arteries were identified and interconnected to establish the central axis of the planned flap. This central axis served as a reference for demarcating the proximal boundary of the wound, oriented towards the exit point of the SCIA. Subsequently, a flap design line was delineated, extending 1.0 cm beyond the periphery of the defect to guide the incision. An incision was meticulously executed proximal to the lateral pulsation point of the femoral artery, followed by a precise dissection to secure the vascular pedicle of the superficial branch of the SCIA. Temporary suturing of adjacent skin was performed to mitigate the risk of tension-induced tearing.

**Fig. 1:**
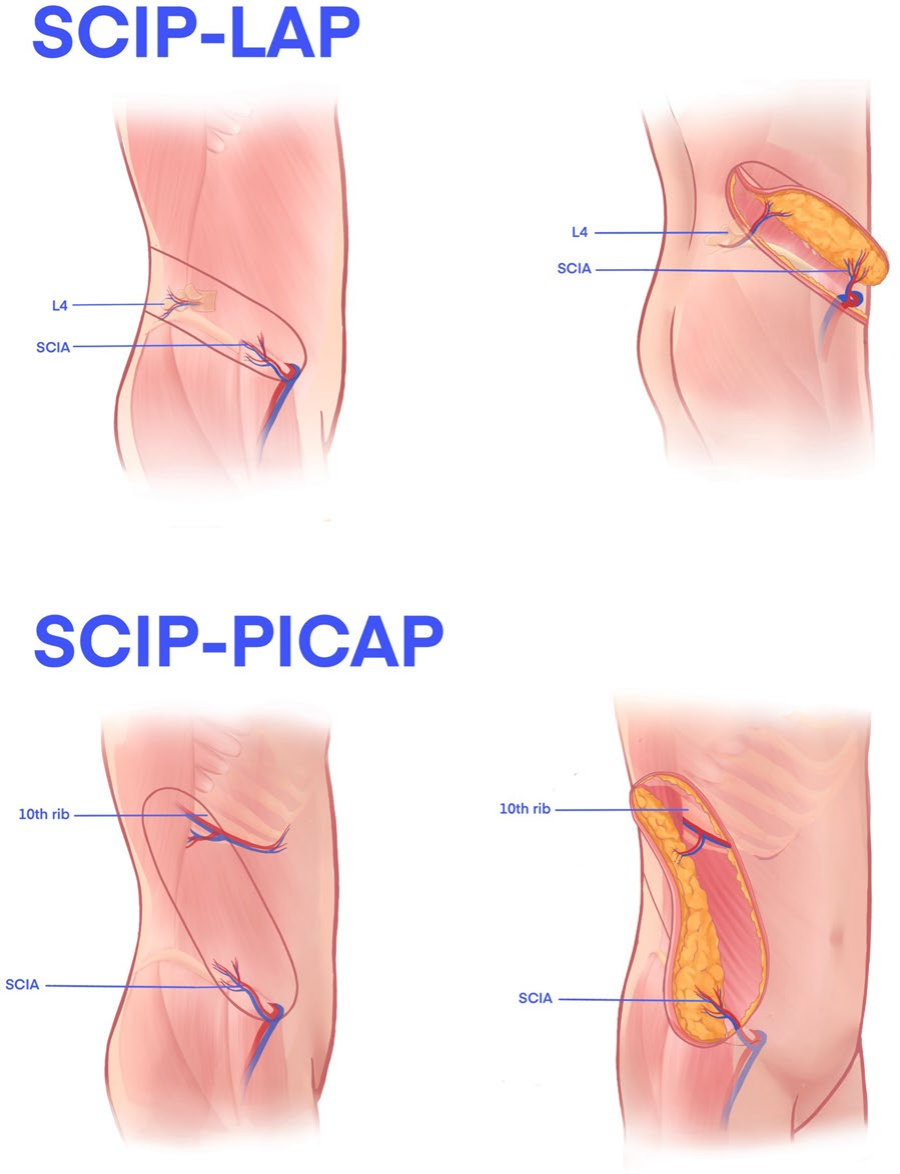
The surgical design schematic

Further incisions were guided either by the mid-axillary line or by the specific markings obtained from the Doppler ultrasound assessment. When the dissection targeted the anterior branch of the L4 artery, it proceeded at the level of the deep fascia to identify and isolate the perforating vessel over a length of 1.0 to 2.0 cm, establishing a reserve vascular pedicle. A similar meticulous dissection approach was employed for the PICA, involving the identification and liberation of the vessel for approximately 2.0 cm to delineate a reserve vascular pedicle. Following complete incision of the skin and securing of the vascular pedicle, the flap was elevated, ensuring adequate blood supply prior to severing the pedicle and harvesting the flap (Fig. 1). Generally, direct closure of the donor site was attainable; however, in instances where direct closure was not feasible, consideration was given to utilizing a full-thickness skin graft obtained from the contralateral site or thigh.

In the meticulous process of venous anastomosis at the proximal extremity of the wound, our exploration unveiled two principal venous networks: those veins in attendance to the superficial branch of the superficial circumflex iliac artery (SCIAs) and the superficial circumflex iliac vein (SCIV) itself. The latter, distinguished by its more substantial girth, assumes the role of the predominant venous egress conduit for the flap. By virtue of its increased diameter, the SCIV emerged as the venous channel of preference for anastomosis. When addressing reconstructions pertinent to upper limb trauma, a direct anastomotic connection was established between the veins escorting the superficial circumflex iliac artery and either the larger subcutaneous veins in proximity to the wound or the venous companions of the brachial artery [9].

Subsequent to the strategic placement of the flap within the designated recipient locale, meticulous alignment was undertaken to ensure that the SCIAs was appropriately juxtaposed with the wound's proximal terminus, and the anterior branch of the L4 artery or the PICA was suitably aligned with the distal terminus. In instances where the vessel's length was deemed insufficient for anastomosis, the crafting of a free vein segment for anastomotic bridging was earnestly considered. Upon the successful execution of the vascular anastomosis, a rigorous verification of the reinstated blood supply and the presence of adequate marginal bleeding was conducted, preceding the insertion of a drainage tube and the subsequent meticulous closure of the skin. In the postoperative phase, patients were counselled to embark upon functional exercises after a fortnight's duration, contingent upon the absence of any contraindications, thereby facilitating a more expedient and effective recovery process.

## Results

Summary of patient characteristics is presented in Table 1. Twelve patients (3 females and 9 males) were included with an average age of 31.7 years (range: 22–44 years). All defects were located in the upper limbs and involved deep tissue (bone or tendon) exposure. The average defect size was 28.8 ± 3.2 × 10.4 ± 1.9 cm, completely covered in all cases. The mean flap size was 30.3 ± 2.7 × 11.6 ± 2.1 cm. Within this case series, due to sufficient pedicle length, no cases required venous bridging, and there were no distal venous anastomoses (Table 2). The mean flap harvesting time was 102.3 ± 11.2 min. To prevent postoperative hematoma complications, all vessels, except those for anastomosis, were ligated intraoperatively. Hemostasis was achieved thoroughly using an electrocoagulation device on bleeding points, followed by drain placement post flap transplantation. In case of hematoma occurrence, medical dressings were changed, and partial sutures were removed. All flaps in the cases survived without complications at the donor sites. Only one case experienced arterial supply disruption due to pressure from a subcutaneous hematoma on the flap, but successful revascularization was achieved after removing some sutures.

All 12 cases were followed up for an average of 15.3 ± 5.6 months.

## Case reports

### Case 1 (Fig. 2)

**Fig. 2:**
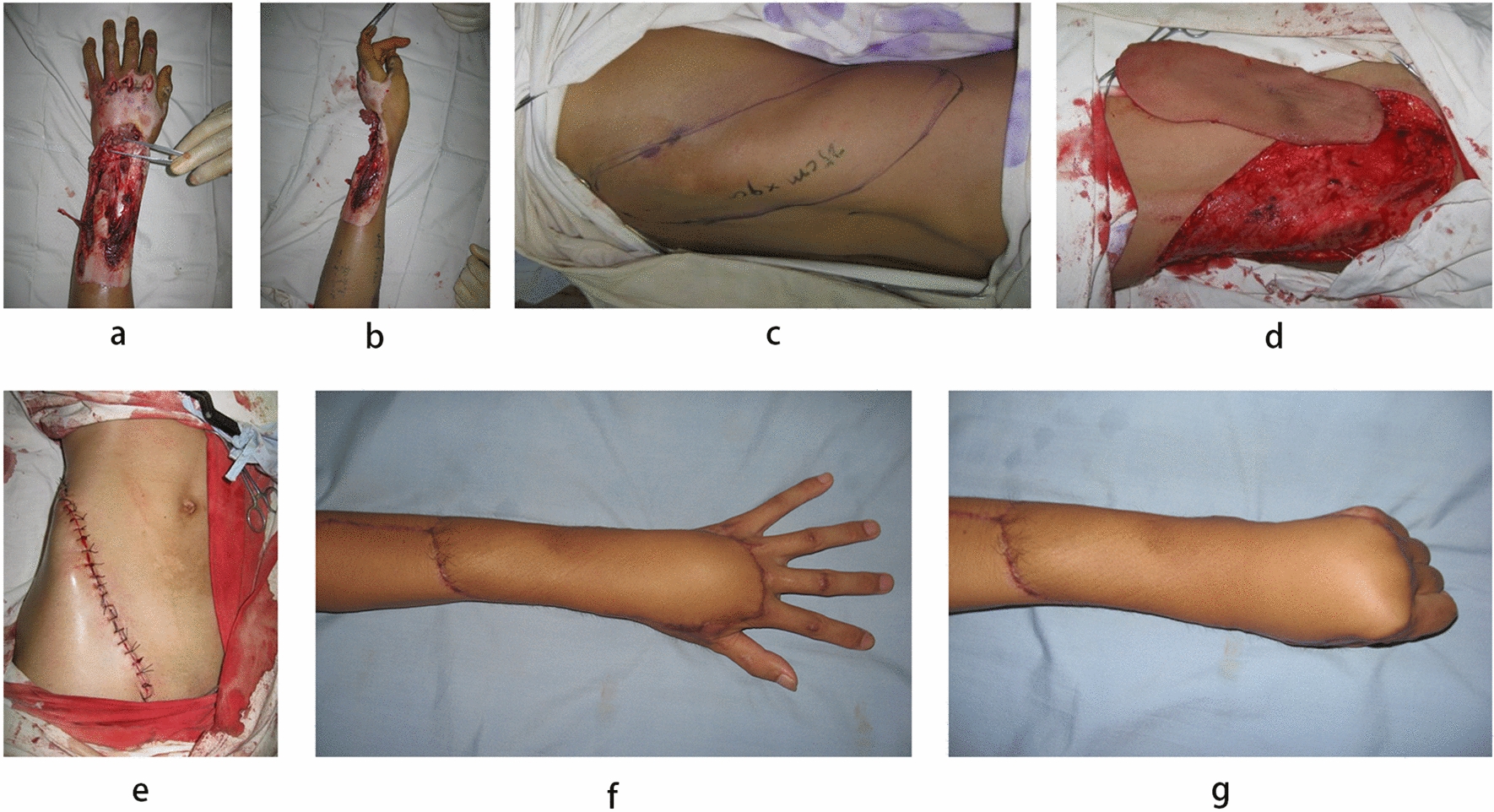
Case 1. a, b Skin defect after debridement. c Locate the anterior branch of the fourth lumbar artery and design the flap. d Flap harvesting and dissection of its perforated pedicle. e Donor area closed immediately. f, g 1 year postoperative result, excellent scar appearance and flexible finger flexion and extension

A 25 year-old male sustained a mechanical injury from a grinding wheel. This resulted in a soft tissue defect extending from the mid-dorsal aspect of the left forearm to the palmar joint of the hand, with an observed defect size of 23 × 8 cm after debridement (Figs. 2a and b). A 25 × 9 cm pedicle flap from the right lower abdomen, incorporating the inguinal and groin regions, was designed (Figs. 2c and d). The proximal end of the flap relied on the superficial branch of the superficial circumflex iliac artery for blood supply, while the distal end relied on the anterior branch of the fourth lumbar artery. Vascular anastomoses: the superficial branch of the superficial circumflex iliac artery anastomosed to the posterior interosseous artery, the companion vein of the superficial circumflex iliac artery anastomosed to the companion vein of the posterior interosseous artery, the superficial circumflex iliac vein anastomosed to the cephalic vein to reestablish the proximal blood supply to the flap, and the anterior branch of the fourth lumbar artery anastomosed to the dorsal metacarpal artery arising from the radial artery. Direct closure was performed at the donor site (Fig. 2e), and the flap survived completely. The patient was discharged after 20 days postoperatively. At the 12 month follow-up, linear scar formation was observed at the donor site, the flap exhibited soft texture, and there was good mobility in the wrist and finger joints. Additionally, the flap displayed sweating function, and protective sensation was restored (Figs. 2f and g).

### Case 2 (Fig. 3)

**Fig. 3:**
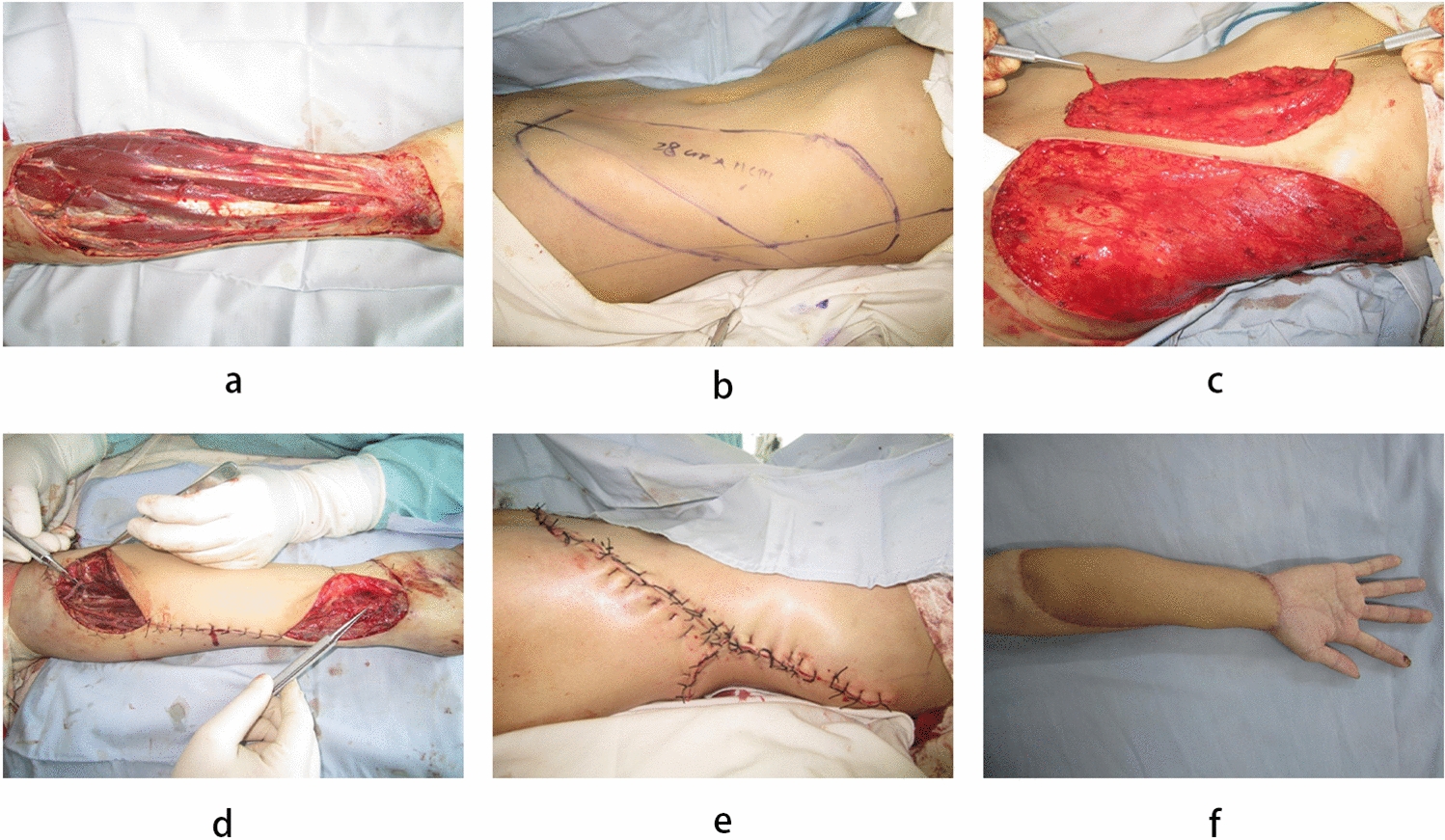
Case 2. a Skin defect after debridement and expansion. b Conjoined perforator flap in the ilioinguinal area based on the lateral perforator of the posterior intercostal artery. c Flap harvesting. d Place the flap and suture the defect area. e Donor Site Direct Closure. f 2 years after surgery, good hand function and cosmetic appearance

A 28 year-old male was admitted to the hospital with a machine-induced thermal crush injury to the right forearm (Fig. 3a). After presurgical wound management, elective surgery was performed 9 days post-injury, revealing a defect size of 26 × 10 cm. A 28 × 11 cm ilioinguinal region syndesmotic perforator flap was designed in the left lower abdomen (Figs. 3b and c). The superficial branch of the superficial circumflex iliac artery was anastomosed to the brachial artery muscular perforator, the accompanying vein of the superficial circumflex iliac artery was anastomosed to the accompanying vein of the brachial artery muscular perforator, the superficial circumflex iliac vein was anastomosed to the subcutaneous vein of the arm, and the lateral branch of the intercostal artery was anastomosed to the superficial palmar branch of the radial artery (Fig. 3d). The donor area was closed directly (Fig. 3e). At the 24 month postoperative follow-up, the right hand exhibited good flexion and extension function and maintained a favorable appearance (Fig. 3f).

## Discussion

The inguinal region, esteemed for its discreet location, substantial skin thickness, and the feasibility of direct suturing, has emerged as an exemplary donor site for skin flaps. This area, favored for its attributes, offers an optimal balance of accessibility and aesthetic discretion, making it a preferred choice among practitioners for the harvesting of skin flaps [6]. Furthermore, the skin flap from the groin region, characterized as an axial pattern flap, presents a more favorable length-to-width ratio, rendering it particularly adept at addressing extensive "long and narrow" skin defects encountered in the upper limbs. This anatomical configuration allows for precise and efficient reconstruction of such specific defect profiles, ensuring a seamless integration and optimal functional and cosmetic outcomes in the upper limb reparative process [9]. Nonetheless, the dependence on merely the SCIAs for vascularization of the skin flap, aimed at reconstructing extensive and disproportionately elongated soft tissue defects, is manifestly insufficient. It is imperative to incorporate additional perforating vessels to guarantee an adequate blood supply crucial for the survival of the flap. Therefore, an approach that encompasses the ilioinguinal flap, tailored specifically to echo the "long and narrow" typology of upper limb injuries and augmented with perforator vessels, assumes considerable clinical importance. This refined strategy enhances the viability and effectiveness of the flap in complex reconstructions, underscoring a pivotal advancement in the domain of microsurgical tissue repair [10].

In the realm of microsurgical advancements, the ilioinguinal flap's vascular architecture is predominantly anchored by two pivotal arteries: the SCIA and the Superficial Inferior Epigastric Artery (SIEA). Our research methodology accentuates the utilization of the superficial branch arteries emanating from the SCIA, which provide a direct vascular pathway to the skin, thereby simplifying their identification on the dermal layer. As the SCIA flap's clinical applications continue to expand, an increasing corpus of evidence has begun to illuminate the adequacy of the arterial pedicle's length and diameter, as derived from the SCIA’s superficial branch arteries, in maintaining the viability of moderately sized flaps. This realization has steered contemporary microsurgical thought towards favoring these superficial branch arteries. They offer a less complex and more accessible alternative to the intricate intramuscular dissections, thereby gaining preferential consideration amongst scholars and practitioners in the microsurgical community [4, 11–14].

In the domain of cutaneous vascularization pertaining to the posterior aspect of the trunk, it is well-documented that the integument receives its blood supply from the PICA, in conjunction with the musculocutaneous ramifications emanating from both the lumbar and lateral sacral arteries. The anatomical canon has, moreover, affirmed the presence of perforators associated with the lumbar arteries, thereby enriching our understanding of the vascular architecture in this region [15]. The lumbar artery perforator (LAP) flap, endowed with a dependable blood supply and ample tissue, is esteemed for its versatility as a flap. Presently, the LAP has been the subject of extensive investigation, particularly as a free flap in the realm of breast reconstruction. The utilization of the fourth lumbar artery emerges as the most prevalent application in this context, illustrating the LAP flap’s significant role in advancing reconstructive surgical techniques [15–17].

In 1979, Kerrigan et al. conducted a detailed study on the course and distribution of the posterior intercostal artery, categorizing it into vertebral, costal, intramuscular, and rectus abdominis segments [18].Badran et al. described the first successfully viable free flap based on the lateral branch of the posterior intercostal artery [19], which opened up new dimensions for reconstructive surgery. Currently, the PICAP flap has been applied in the repair of a few large-area back defects [20].

The management of extensive skin deficits necessitates the exploration and clinical application of various adaptations of the SCIP flap. Hong et al. have elucidated the technique for harvesting substantial flaps (measuring 12 × 32 cm) using both superficial and deep perforators from the SCIA, with an average vascular pedicle length of 5 cm observed in their study subjects. Moreover, they have highlighted the anatomical variability present in the deep perforators of the SCIP flap and the imperative for supermicrosurgical techniques to facilitate their effective utilization [21]. Concurrently, Yamamoto et al. have successfully employed both superficial and deep perforators of the SCIA to augment the SCIP flap, creating a large flap (23 × 15 cm) intended for breast reconstruction, which necessitated a considerable volume of tissue [22].

The harvesting of deep perforators inherently encompasses a greater tissue volume compared to their superficial counterparts. Within these referenced studies, there was a discernible demand for flap volume; however, for defects restricted to superficial skin and soft tissues, such extensive tissue volumes prove unnecessary, potentially necessitating secondary debulking procedures.

Fernandez-Garrido and colleagues have innovatively extended the SCIP flap towards the cranial region, based on the SCIA trunk. This approach not only facilitated the procurement of expansive flap areas but also enhanced the length and diameter of the vascular pedicle [23].However, the retrieval of deep perforators or the SCIA trunk demands intricate and deeper anatomical dissections, significantly increasing the procedural complexity and duration, while elevating the risk of intra-abdominal injury and enhancing donor-site morbidity. In light of the anatomical variability of deep perforators and to diminish the dissection complexity, extensive literature recommends the preoperative localization of perforating vessels via CT angiography (CTA). Although not obligatory, this practice is broadly advocated. Due to our equipment limitations, employing CTA for preoperative localization is unfeasible. Therefore, selecting more superficial perforators, which are less affected by anatomical variability, represents a more prudent and efficient strategy.

Choosing deep perforators or the SCIA trunk to obtain a pedicle with relatively greater length, in comparison to superficial branches, might lead to vascular tortuosity in transplants intended for upper limb soft tissue defects, suggesting that an elongated pedicle does not uniformly confer a therapeutic benefit.

Yamamoto et al. have also demonstrated the procurement of extensive composite flaps (20 × 20 cm) by combining the SCIP flap with flaps from the superficial inferior epigastric artery (SIEA) or the deep inferior epigastric artery perforator (DIEP) flaps. Although this strategy exemplifies an excellent design for acquiring large flap areas, the disparate vascular pathways do not align on a common axis, inadequately catering to the repair needs of elongated soft tissue defects with significant length-to-width ratios in the upper limb [24].

In scenarios characterized by limited equipment availability or insufficient experience in supermicrosurgical techniques, for upper limb soft tissue defects described as "long and narrow," the strategic utilization of the anatomical positioning, expandability, and minimally invasive nature of the SCIP, LAP, and PICAP flaps can be employed to design conjoined perforator flaps that are more appropriately tailored. Conjoined perforator flaps, serving as composite flaps, play an irreplaceable role in the design of extensive flaps or the repair of multidimensional defects. They offer significant advantages by obviating the risks associated with multiple flap junction scars and flap wound dehiscence, thereby reducing morbidity at the donor site and diminishing the duration and intensity of surgical endeavors [25–27].

The augmentation of blood flow to the flap, thereby broadening the scope for flap harvesting, can be achieved through the synergistic combination of the SCIA with either the L4 artery or the PICA, facilitating external pressure upon the flap. Orienting the flap along the longitudinal axis of the trunk permits the direct closure and suturing of the donor site, attributable to the reduced soft tissue thickness prevalent along the lateral aspect of the trunk. This strategic design not only optimizes the use of available tissue but also diminishes the necessity for secondary thinning procedures. Within this series, a mere duo of cases presented with a Body Mass Index (BMI) surpassing the threshold of 24, necessitating the execution of a secondary thinning operation subsequent to the transplantation of the SCIP-LAP combined flap, with the aim of meeting aesthetic criteria for the appearance of the upper limb.

This investigation delineated two methodologies for the procurement of compound SCIP flaps: (1) the engagement of the SCIAs conjoined with the anterior branch of the L4 artery; (2) the incorporation of the SCIAs in tandem with the PICA. Our avant-garde approach to the ilioinguinal region employing the composite perforator flap has markedly expanded the expanse of flap area amenable to harvesting. Within the confines of this study, the most extensive flap area successfully sustained was 35.0 × 15.0 cm, showcasing robust blood perfusion. Intraoperatively, the observation of active bleeding along the periphery of the excised flap served as a testament to the adequacy of the vascular supply, sufficing to nurture the entirety of the flap. Notably, this investigation recorded no instances of injury to the principal arterial conduit in the donor zone. The incidence of morbidity was minimal, with a complete survival of all flaps, heralding an optimistic prognosis. Pertinently, this surgical modality obviated the necessity for venous anastomosis at the wound's distal extremity, circumventing the escalation of venous return pressure within the flap [28].

Within this series of cases, multiple perforating vessels were identified at the flank and lower back regions, offering a variety of options for selection. The perforators emanating from both the lumbar arteries and the PICA are readily approachable in a subcutaneous manner, thus enabling expeditious and uncomplicated anatomical dissection, as delineated in the supplemental digital content provided (Additional file 1).

Owing to the constricted corpus of cases, the scope and gravity of surgical complications inherent to this compound SCIP flap design remain incompletely understood. Consequently, a more exhaustive and forward-looking assessment across a broader demographic is imperative to gain a fuller appreciation of its efficacy and safety profile. Furthermore, the election to utilize the lateral perforating branch of the PICA entails that the flap's donor site may only incorporate a segment of the lateral cutaneous branch of the intercostal nerve, thereby circumscribing its innervation potential. This limitation necessitates careful consideration in the planning phase to optimize functional and aesthetic outcomes.

The employment of a free ilioinguinal flap in conjunction with the SCIAs, the anterior branch of the fourth lumbar artery, or the PICA can significantly amplify the harvestable expanse of the flap while guaranteeing a substantial blood supply. This particular flap configuration is distinguished by its ease of harvest, dependable perforators, slender texture, and the feasibility of direct donor site closure. It embodies an innovative and optimal strategy for the one-stage reconstruction of extensive and slender soft tissue deficits in the upper limb, presenting a paradigm shift in reconstructive surgery techniques.

## Conclusions

The compound SCIP flap design enhances the utility of the axis-oriented flap emanating from the ilioinguinal region, thereby augmenting skin availability. This methodology introduces an additional donor site alternative for one-stage transplantation, adeptly addressing the challenge of repairing extensive "long and narrow" skin defects on the upper limb. It simultaneously mitigates the likelihood of additional surgical scarring and the risk of wound dehiscence. For the surgical practitioner, this design promises simplified access to larger flaps, diminishing the intricacy of the surgical procedure and curtailing the duration of operation. In light of these merits, it is advisable to regard the compound SCIA flap from the ilioinguinal region as a viable and effective solution for the reconstruction of extensive "long and narrow" skin defects on the upper limb.

### Supplementary Information

**Additional file 1: **A 31 year-old male underwent a SCIP-PICAP (Superficial Circumflex Iliac Artery Perforator-Profunda Artery Perforator) procedure for the reconstruction of soft tissue defects in the upper extremity, with evaluation of the anatomy of intercostal posterior artery perforators

## Data Availability

All data published here are under the consent for publication.

## Abbreviations

L4 artery: The fourth lumbar artery
LAP: Lumbar artery perforator flap
PICA: Posterior intercostal artery
PICAP: Posterior intercostal arteries perforator
SCIP: Superficial circumflex iliac artery perforator flap
SCIA: Superficial circumflex iliac artery
SCIAs: Superficial branch of superficial circumflex iliac artery
AV-SCIA: Accompanying vein of the superficial branch of the circumflex iliac artery
SCIV: Superficial circumflex iliac vein
PIA: Posterior interosseous artery
PIAV: Vein accompanying the posterior interosseous artery
RA: Radial artery
SBRA: Superficial branch of the radial artery
BA: Brachial artery
AV-BA: Accompanying vein of the brachial artery
CV: Cephalic vein
SD: Standard deviation
