# Supplementary figures and images for: Repair of a “long and narrow” skin defect of the upper extremity with a modified design of a compound SCIP flap: a series of 12 cases
Source: Eur J Med Res. 2024 May 9;29:275. doi: 10.1186/s40001-024-01863-y (PMC11080178; doi:10.1186/s40001-024-01863-y)

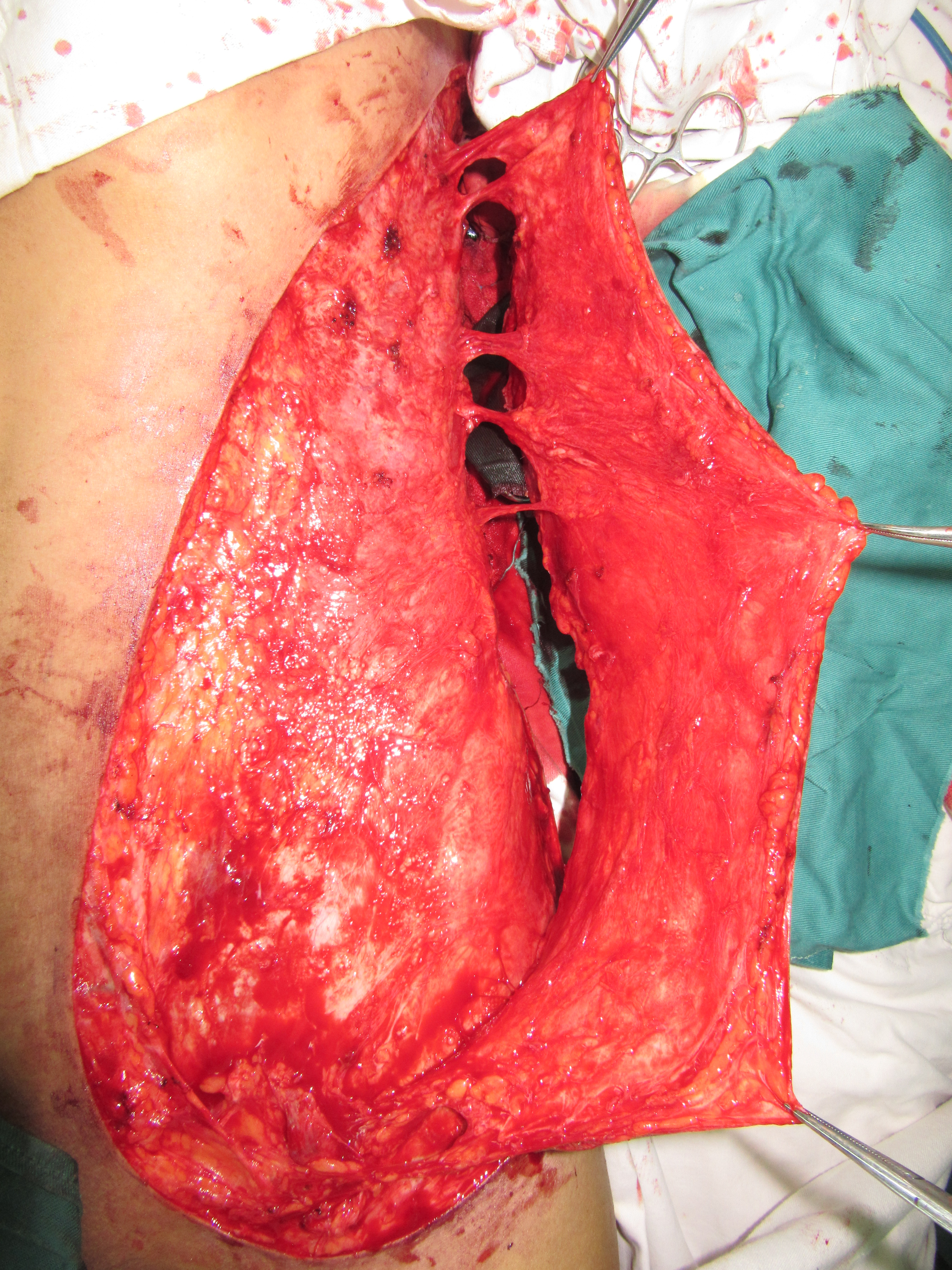

Supplement: Supplementary file 1 — Additional file 1: A 31 year-old male underwent a SCIP-PICAP (Superficial Circumflex Iliac Artery Perforator-Profunda Artery Perforator) procedure for the reconstruction of soft tissue defects in the upper extremity, with evaluation of the anatomy of intercostal posterior artery perforators [file 40001_2024_1863_MOESM1_ESM.tiff]
